# Ameliorative effects of date palm kernel extract against fenpropathrin induced male reproductive toxicity

**DOI:** 10.1186/s40659-025-00605-6

**Published:** 2025-05-06

**Authors:** Maher M. Soliman, Marsail S. Nashed, Eman I. Hassanen, Marwa Y. Issa, Abdelbary M. Prince, Ahmed M. Hussien, Adel F. Tohamy

**Affiliations:** 1 Department of Toxicology and Forensic Medicine, Faculty of Veterinary Medicine, Cairo University, Giza, Egypt; 2 Department of Pathology, Faculty of Veterinary Medicine, Cairo University, Giza, Egypt; 3 Department of Pharmacognosy, Faculty of Pharmacy, Cairo University, Giza, Egypt; 4 Department of Biochemistry and Molecular Biology, Faculty of Veterinary Medicine, Cairo University, Giza, Egypt

**Keywords:** *Date palm kernel*, Fenpropathrin, Testosterone, Oxidative stress, Caspase 3, StAR gene

## Abstract

**Background:**

The purpose of this work was to examine the fundamental mechanisms of reproductive toxicity in rat models following exposure to Fenpropathrin (FNP). Furthermore, our study explores the novel impacts of Date palm kernel extract (DPK) on these detrimental outcomes.

**Methods:**

Thirty male Wistar rats were used in the investigation. They were split into six groups: one group received corn oil as a control; two groups received DPK at 200 mg/kg and 400 mg/kg; a group received FNP at 4.7 mg/kg; and two combination groups received DPK and FNP at 200 mg/kg and 400 mg/kg, respectively for 60 days.

**Results:**

FNP caused oxidative stress, reduced sperm count, and impaired motility. FNP decreased the expression of the StAR gene and reduced serum testosterone levels. We assessed the histological alterations. In a dose-dependent way, the concurrent administration of DPK extract successfully decreased all the toxicological parameters.

**Conclusions:**

When taken orally, DPK extract may protect against FNP-induced male reproductive toxicity.

## Graphical Abstract

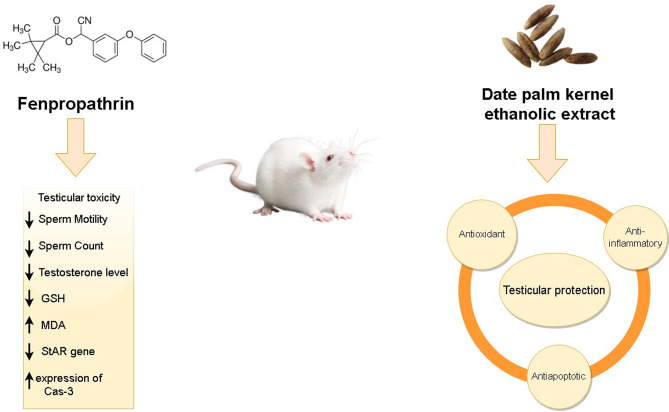

## Background

Synthetic pesticide use in agriculture has negatively impacted rural ecosystems [1, 2]. Numerous negative impacts have been brought about by the discharge of these substances into the environment [3]. Both wealthy and developing countries experience health problems for people and animals because of these pollutants’ effects on the land and water [4].

Fenpropathrin (FNP) is a member of the α-cyano group pyrethroids (PYRs) class, also called to as 3-phenoxy benzyl-2, 2, 3, and 3-tetramethyl cyclopropane carboxylate. FNP has a high degree of absorption in organic materials, soils, and suspended particles in the water column. This is mainly explained by its nonpolar properties, strong solubility in other organic solvents, and limited solubility in water [5]. According to Mohamed et al. [6], FNP exhibits a noteworthy propensity to bioaccumulate in organisms. FNP possesses a robust control mechanism, a long-lasting effect, a broad insecticidal spectrum, and light stability. Low-level exposure to FNP damages lymphatic cells, the neurological system, the liver, the kidneys, and the reproductive system [7]. Zeid et al. [5] state that FNP can modify mitochondria size by reducing the dynein expression level associated with mitochondria. It can produce highly reactive metabolites and reactive oxygen species (ROS) in addition to FNP, which can cause oxidative stress [8]. Jebur et al. [9] discovered that FNP-treated rats had more testicular thiobarbituric acid-reactive chemicals, hydrogen peroxide, protein carbonyls, and aminotransferases and phosphatases. The study found significant decreases in body weight, gonadosomatic index, glutathione levels, protein content, enzymatic antioxidants, and hydroxysteroid dehydrogenases (3β and 17β HSD).

The hard, elongated form of date palm kernel seeds, also known as DPK, is distinguished by ventral grooves. Date seeds are frequently found inside the date palm tree’s (Phoenix dactylifera) fruit. Date palms are primarily grown in semi-arid and dry climates, like Africa, the Arabian Peninsula, and the United States. It’s vital to remember that the selling of date palms provides a substantial amount of wealth for people living in the Sahara region [10]. In addition, it was projected that 8.5 million tons of date fruits were produced in 2016 [11]. The Date palm Kernel (DPK) makes up around 10% of the entire mass of the whole date fruit. As a result, the production of date seed was anticipated to be 850 thousand tons in 2016.

Numerous research has confirmed the nutritional significance of palm date seeds, particularly their antioxidant content. It was believed that the specimens included significantly more high-quality biological protein and fat than date flesh. Furthermore, DPK’s high phenolic and dietary Fiber content may be responsible for their advantages, making them a crucial component in preparing functional meals [12]. According Attia et al. [13] date palm seed fatty acids are: Lauric acid is 12,200–23,060 mg / 100 g, Myristic acid is 9,700–11,300 mg / 100 g, Palmitic acid is 10,110–12,700 mg / 100 g, Stearic acid is 1,560–3,560 mg / 100 g, Between 35,100 and 45,800 mg / 100 g is oleic acid, Linoleic acid is 8,100–11,000 mg / 100 g, and Linolenic acid is 370–800 mg / 100 g. Date seeds have higher total polyphenols than other fruits [14]. Date seeds contain seven phenolic acids: caffeic, chlorogenic, p-coumaric, ferulic, gallic, syringic, and vanillic. P-coumaric acid has the most phenolic acids, 109.87–141.72 mg/100 g [15]. Rutin is the main flavonoid in date seeds, with concentrations of 71.74 to 86.32 mg / 100 g, according to HPLC results with a diode-array detector (DAD). Quercetin is 23.71–34.06 mg / 100 g, while luteolin is 9.17–13.24 mg / 100 g.

The extensive use of FNP for pest management causes it to bioaccumulate in the environment, making exposure to people and animals possible. On the other hand, using natural plant-based treatments might reduce the danger related to environmental pollution. Nevertheless, there has yet to be any mention of the date palm kernel’s interaction with FNP. Therefore, by monitoring the changes in the testosterone hormonal analysis, this study sought to determine if Date palm kernel extract (DPK) might protect male rats from FNP-induced infertility and reproductive dysfunction.

## Materials and methods

### Chemicals

Sumitomo Chemical Co., Ltd. in Japan provided the commercial form of FNP (20%, emulsifiable concentrate (EC), chemical formula 3-phenoxy benzyl-2, 2, 3, 3-tetramethyl cyclopropane carboxylate).

### Animals

Thirty healthy male Wistar rats, weighing 180 ± 20 g at 12 weeks of age, were purchased for the experiment from the Research Institute of Ophthalmology in Giza, Egypt. The rats were housed in a room with good ventilation, 24 °C temperature, and 60% relative humidity. They had ad libitum diet and were kept in a plastic cage. Before participating in the study, the animals were given two weeks to get acquainted with the laboratory environment. The institutional animal care and use committee (Vet. Cu. IACUC) at the College of Veterinary Medicine, Cairo University, Giza, Egypt, has accepted the experimental design (approval number: 03162023654). All procedures involving animals were conducted in compliance with the guidelines set by the National Institutes of Health (NIH) Guide for the Care and Use of Laboratory Animals [16, 17].

Animals per group were selected according to Arifin et al. [18] following equation: n = (DF /K) + 1.

### Plant extraction

In September 2022, three kilograms of roasted date palm kernel seeds (*Phoenix dactylifera L.*) were bought from Aswan. The roasted seeds were ground into a fine powder using a mechanical herb grinder. At room temperature, 70% ethanol was used in consecutive extractions of the powdered substance. The reddish-brown residue obtained from the extraction process weighed 252 g in total. The material was stored at -20 °C until analysis and biological activity assessment.

### Sample preparation for ultra-performance liquid chromatography-mass spectrometry (UPLC-ESI-QTOF-MS) analysis

The sample was analyzed at the Children’s Cancer Hospital 57,357, Basic Research Department, Proteomics and Metabolomics Research Program. A 50:25:25 combination of acetonitrile reconstitution solvent, methanol, and water dissolved date palm kernel ethanolic extract. A 50-milligram extract solution required one millilitre of solvent. After vortexing, the solution was ultrasonicated. The mixture was centrifuged for 10 min at 10,000 RPM. 50 µl and 1000 µl of reconstitution solvent were used to create the standard solution. The final concentration reached 2.5 µg/µl. An aliquot of 10 µl of solution was placed in negative mode. No excess material was employed, and just a 10 µl reconstitution solvent sample was used [8].

### Analysis with high-resolution UPLC-ESI–QTOF-MS

The mobile phase consisted of two components. Mobile phase B was 100% pure acetonitrile, while phase A was 5 mM ammonium formate buffer with 1% (v/v) methanol and pH 8. This experiment employed linear gradient elution. Rules for elution included: Initial solution was 90% solvent A and 10% solvent B. After 21 min, solvent A was 10% and B 90%. After then, the eluent was changed to 90% solvent A and 10% solvent B for 25–28 min. This study utilized a 0.5 μm × 3.0 mm in-line filter disc from Phenomenex^®^ in Torrance, CA, USA, as the pre-column. The primary column used was a Waters^®^ XBridge C18 column (3.5 μm × 2.1 × 50 mm) from Milford, Massachusetts, USA. The column was heated to 40 °C and flowed 0.3 mL/min. Additionally, a 10 µL injection volume was used. After modifying acquisition settings, LC-QTOF with negative TOF MS ran for 28 min, 0.6502 s, and 2584 cycles. MS1 TOF mass calibration was 50–1000 Daltons. The scanner was HR-TOF. The value is 45 for GS1 and GS2. We have 25 current capillary units. The temperature was 500 units and ISVF was − 4500 units. Information-dependent acquisition (IDA) scan modality was used to calibrate MS2 acquisition; TOF mass measurements were limited to 50.0000–1000.0000 Da; DP, CE, and CES were 80, -35, and 15, respectively [19].

### Experimental design

Six groups of five rats apiece were randomly assigned. The rats were given the specific ingredients orally by gavage daily for 60 days. Group [1] was administered corn oil and served as the control group. Group [2] was administered DPK at 200 mg/kg of body weight. Group [3] was administered DPK at 400 mg/kg body weight [20]. Group [4] was administered FNP at a concentration of 4.7 mg/kg body weight, which is equivalent to 1/15 of the lethal dose 50 (LD_50_) dissolved in corn oil [21]. Groups 5 and 6 were administered FNP and DPK at 200 and 400 mg/kg, respectively.

After the sixty-day experiment, blood samples were obtained from the retro-orbital plexus. Clear serum samples that were centrifuged for 20 min at 3000 rpm were kept at -20 °C until the testosterone levels were determined. The rats were euthanized via cervical dislocation. The testes were dissected right away, and as previously mentioned by Habib et al. [22], sperm were extracted from the cauda epididymis. The testes were split into two sections: the first was fixed in 10% neutral buffered formalin for histopathological and immunohistochemical investigation, and the second was stored at -80 °C until it was needed for molecular research and oxidative stress assessment.

### Sperm analysis

Each rat’s seminal material was extracted by carefully squeezing it in a sterile watch glass after the cauda epididymis’ tail was surgically severed [23]. The material was diluted with a 2.9% sodium citrate solution, a factor of ten. After that, the diluted solution was thoroughly mixed to ascertain the sperm count and the proportion of sperm with progressive motility. Using a light microscope with a 40× objective lens and a haemocytometer, this was carried out using the method outlined by Bearden and Fuquay [24].

### Serum testosterone

A competitive ELISA (Rat) kit from Biovision Egypt Co., Giza, Egypt, was used to estimate serum testosterone using the method outlined by Demetrious et al. [25].

### Oxidative stress markers

According to Ismail et al. [26], samples of frozen testicular tissues were well combined with a cold buffer. The assessment of lipid peroxidation was then carried out by spectrophotometrically determining the concentrations of MDA at 534 nm. Using kits from the manufacturer (Biodiagnostic Com, Cairo, Egypt), the amount of the antioxidant GSH was measured at a wavelength of 405 nm.

### Quantitative real-time polymerase chain reaction analysis for star gene

Total RNA was extracted utilizing Trizol (Invitrogen) according to the manufacturer’s instructions. cDNA was synthesized utilizing M-MuLV Reverse Transcriptase (NEB#M0253) according to the specified technique. The following primer sequences were used in the experiment for the StAR gene: 5′-AGCTCCAAATGCCACTACCT-3′ is the forward primer sequence, and 5′-TGGCCTTTTACAGAGGAGCA-3′ is the reverse primer sequence. We extracted RNA that was enriched with small RNA species to perform mRNA quantitative reverse transcriptase PCR. This was accomplished by utilizing an RNA isolation kit and adhering to the manufacturer’s (Ambion, Austin, TX, USA) instructions. Reverse transcription was performed on the mRNA using first-strand complementary DNA synthesis kits that are available for purchase (Invitrogen). Using a CFX96 instrument made in the United States by Bio-Rad, the experimental method used quantitative reverse transcriptase PCR (qRT-PCR) with Power SYBR Green PCR Master Mix. A heat treatment was applied to the cDNA samples for three minutes at 94 °C. After that, 45 iterations in all were carried out, with each iteration consisting of 15 s at 94 °C, 10 s at 57 °C, and 30 s at 72 °C. According to Rizk et al. [27], the relative quantity approach was used to analyze the data.

### Histopathological examination

After being preserved for 48 h in 10% neutral buffered formalin, the gathered testicular tissue specimens were processed using the conventional technique outlined by Bancroft and Gamble [28]. In short, wax specimens were soaked in paraffin wax, cut into slices that were 4.5 microns thick, purified using xylene, and dehydrated using graded alcohol (70–100%). Using a BX43 light Olympus microscope, all sections were stained with hematoxylin and eosin (H&E) and examined for any histological alterations. An Olympus DP27 digital camera connected to the CellSens dimensions program was used to capture our photos.

The degree of testicular lesions in the various experimental groups was evaluated using a simple classical scoring system, following the methodology outlined by Azouz and Hassanen [29]. The primary histological parameters considered in such a scoring system were tubular edema, luminal desquamation, seminiferous tubular atrophy, and germinal cell degeneration and necrosis. The percentage of the affected ST relative to the total number of tubules was measured using grading scales ranging from 1 to 5, with the categories being (1) < 10% (2), 10–25% (3), 25–50% (4), 50–75%, and (5) > 75% ST change. In addition to tubular scoring, a semiquantitative 6-point grading scale was used to examine Leydig cell (LC) number as follows: Normal LC count is between three and five% in every field; moderate LC count decline is less than 3% per field; loss of LC occurs; in contrast, there are four different levels of LC hyperplasia: mild, moderate, and severe, with nodular formation in the sixth level [30].

### Testicular caspase-3 localization

Testicular tissue sections that had been dried and deparaffinized were treated with a 1/200 dilution of the casp-3 primary antibody (Abcam, Ltd.). They were then treated with the secondary antibody and the necessary substrates (Power-Stain 1.0 Poly HRP DAB Kit; Sakura) for the antigen-antibody immunological reaction. Ultimately, the sections underwent examination using an Olympus BX43 light microscope after being counterstained with hematoxylin to determine the level of immunopositivity in each experimental group. The proportion of immunopositive cells to all target cells, including germ cells and interstitial Leydig cells, was calculated using Image J software.

### Statistical analysis

Using the SPSS 27.0 software’s one-way analysis of variance (ANOVA) function, the study’s data were statistically analyzed. The significance between the various experimental groups was then evaluated using Turkey’s honestly significant difference (HSD) test for multiple comparisons. It was considered that a probability level of less than 0.05 was statistically significant. The standard error of the mean (SEM) was displayed alongside the mean value in the data. GraphPad Prism 8, a program created in San Diego, California, USA, by GraphPad Software Inc., was used to make the graphs used in this investigation.

## Results

### Metabolites profile of date palm seeds via UPLC‑MS/MS

Using UHPLC-MS2 in negative ionization mode (ESI), the metabolites from the hydroethanolic extract of Date palm kernels were analyzed. A few classes of metabolites contained in the sample were identified with the help of the analysis, which involved the identification of molecular ions corresponding to [M-H]- and other fragment ions with lower m/z values.

**Fig. 1:**
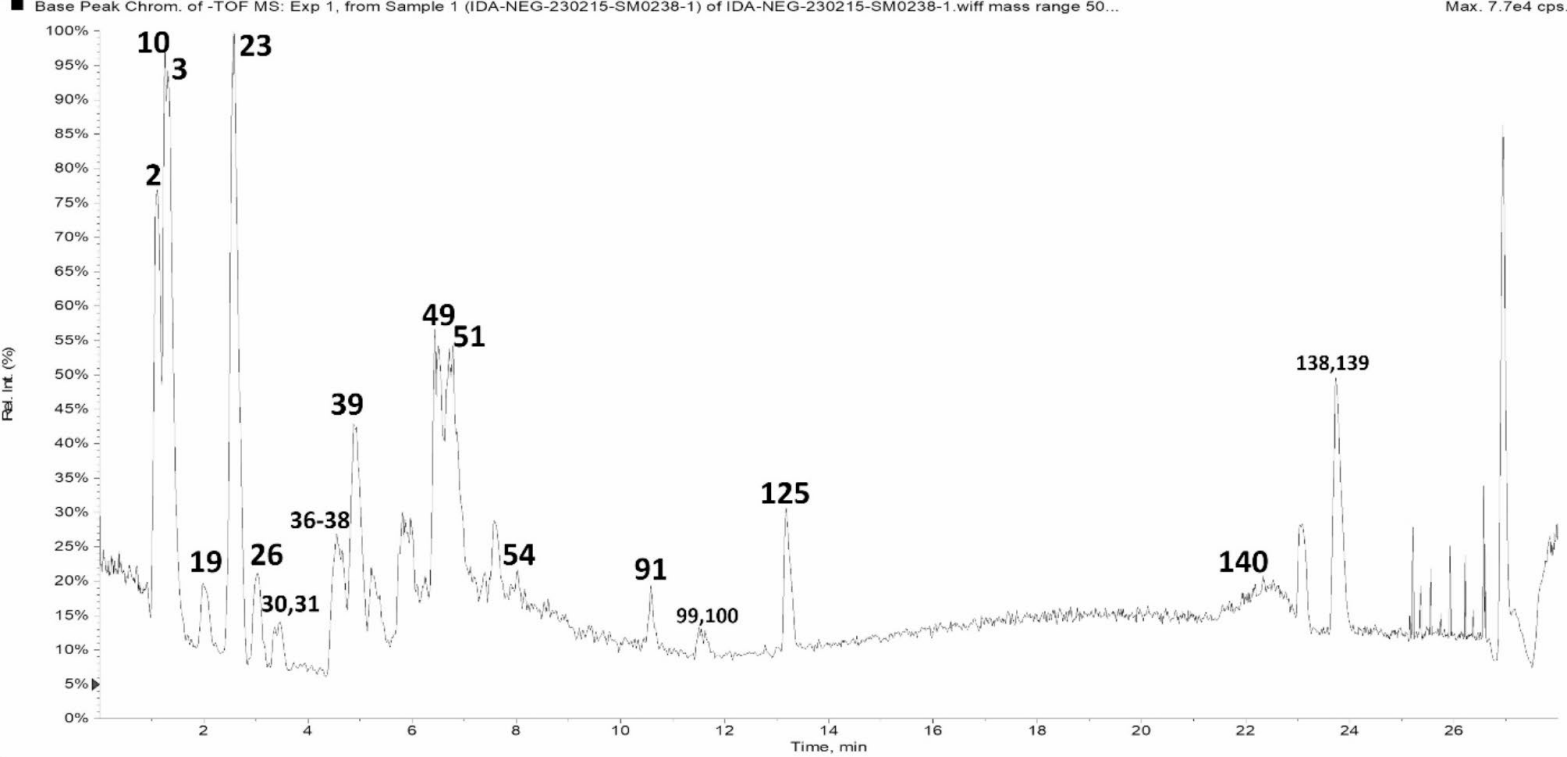
Representative UPLC-MS base peak chromatogram of ethanol extract of date palm seeds in negative ionization mode

169 metabolites in the negative ion mode were identified and categorized using the approach from the date palm kernel ethanol extract. The metabolites include 22 fatty acids, 8 organic acids, 10 coumarins, 4 stilbenes, 66 phenolic acids and their derivatives, 47 flavonoids, and their derivatives, and 12 miscellaneous compounds (Table 1; Fig. 1).

**Table 1:** Identification of phytochemical compounds in date palm seeds ethanol extract by UPLC-ESI–QTOF-MS analysis

Peak	Assignment	RT (min.)	Precursor ion (m/z)	Error (ppm)	Molecular Formula	MS (m/z)
*Organic acids*						
1.	Succinic acid	1.02	117.0198	4.27	C4H6O4	100,73
2.	Glyceric acid	1.09	105.0193	-0.30	C3H6O4	73,61
3.	Itaconic acid	1.31	129.0207	8.52	C5H6O4	111,85,59
4.	Itaconic acid isomer	1.59	129.0191	-1.55	C5H6O4	85,69,59
5.	Isopropylmalic acid	1.64	175.617	2.87	C7H12O5	131,87
6.	Citramalic acid	2.83	147.0301	1.38	C5H8O5	129,103,85
7.	Cinnamoyl hexose	5.18	309.0987	2.34	C15H18O7	247,241,188,147,137
8.	Cinnamic acid	7.96	147.0444	-4.76	C9H8O2	119,103,90,69
*Phenolic acids and derivatives*						
9.	Gallic acid	0.45	169.0150	4.73	C7H6O5	133,125,101
10.	Ferulic acid	1.30	193.0504	-1.04	C10H10O4	175,149,123
11.	Vanillic acid	1.54	167.0345	-2.99	C8H8O4	152,123,108
12.	Dihydroxybenzoic acid	1.69	153.0191	-1.31	C7H6O4	109,81,65
13.	Syringic acid	1.76	197.0467	5.85	C9H10O5	179,151,135,123
14.	*p*-Coumaric acid	1.79	163.0402	1.23	C9H8O3	119,95,77
15.	Dihydro-ferulic acid	1.82	195.0666	2.05	C10H12O4	177,151,136,123
16.	Caffeic acid	1.85	179.0358	4.57	C9H8O4	151,135,117,99,77
17.	Gallic acid	1.99	169.0152	5.64	C7H6O5	125,101
18.	Sinapic acid	2.01	223.0617	2.68	C11H12O5	179,135,91,59
19.	Caffeoylquinic acid	2.04	353.0872	-1.72	C16H18O9	307,289,191,179,135
20.	Dihydro-ferulic acid	2.12	195.0662	-0.42	C10H12O4	151,123,109
21.	Caffeic acid ethyl ester	2.16	207.0682	9.26	C11H12O4	179,163,119, 109
22.	Hydroxy benzoic acid hexoside	2.24	299.0780	2.54	C13H16O8	253,241,187,175,147,125
23.	Ferulic acid isomer	2.58	193.0506	-0.17	C10H10O4	149,121
24.	Caffeic acid isomer	2.60	179.0358	4.57	C9H8O4	151,135,117,99,77
25.	Caffeic acid ethyl ester	2.68	207.0664	0.57	C11H12O4	179,163,119,109
26.	Dihydroxybenzoic acid isomer	3.01	153.0198	3.26	C7H6O4	123,113,109,85,77
27.	Vanillin	3.10	151.0402	0.87	C8H8O3	108
28.	Dihydroxybenzoic acid isomer	3.34	153.0194	0.44	C7H6O4	123,113,109,85,77
29.	Dihydro-ferulic acid	3.35	195.0651	-5.63	C10H12O4	177,151,122,109
30.	Dihydro-ferulic acid	3.39	195.0661	-0.51	C10H12O4	177,151,122,108
31.	Dihydro p-coumaric acid	3.43	165.0559	1.10	C9H10O3	137,123
32.	Dihydro-ferulic acid	3.54	195.0653	-4.61	C10H12O4	177,159,151,122
33.	Gallic acid	3.85	169.0150	4.73	C7H6O5	151,137,125
34.	Dihydroxybenzoic acid isomer	3.86	153.0198	3.26	C7H6O4	109,83,77
35.	Dihydroxybenzoic acid isomer	4.03	153.0186	-4.57	C7H6O4	107,83,65
36.	Syringic acid	4.58	197.0454	-0.75	C9H10O5	179,161,153,135,113,85,79
37.	Vanillic acid	4.64	167.0356	3.70	C8H8O4	149, 123, 108
38.	Dihydroxybenzoic acid isomer	4.65	153.0186	-4.57	C7H6O4	135,123,107,77
39.	Coumaroyl malic acid	4.81	279.0516	2.57	C13H12O7	235,199,176,133,103
40.	Dihydro coumaric acid	4.95	165.0577	7.87	C9H10O3	137,123,95,77
41.	Hydroxybenzoic acid	5.22	137.0251	5.10	C7H6O3	109,81
42.	Syringic acid	5.25	197.0441	-7.10	C9H10O5	153,113,85,79
43.	Coumaroyl glycolic acid	5.29	221.0443	-5.64	C11H10O5	191,177,163,135,113
44.	P-hydroxybenzoic acid	5.62	137.0240	-2.91	C7H6O3	108,92
45.	4-Vinylsyringol	5.74	241.0855	-6.30	C15H14O3	197,183,179,155,137
46.	Hydroxybenzoic acid	5.87	137.0240	-2.91	C7H6O3	108,92
47.	Dihydrocaffeic acid	6.00	181.0516	5.35	C9H10O4	137,123,113
48.	Hydroxy-phenylacetic acid	6.31	151.0405	3.31	C8H8O3	136,108
49.	p-Coumaric acid	6.59	163.0399	-0.61	C9H8O3	135,95,77
50.	Caffeic acid	7.13	179.0358	5.02	C9H8O4	135,99,77
51.	Vanillic acid	7.17	167.0356	4.19	C8H8O4	152,123,95,93
52.	Ferulic acid	7.81	193.0501	-2.58	C10H10O4	149,121,93
53.	Hydroxybenzoic acid	7.93	137.0239	-3.64	C7H6O3	108,93,91
54.	Ferulic acid	8.05	193.0504	-1.03	C10H10O4	177,149,121,93
55.	Dihydro-ferulic acid	8.19	195.0650	-6.15	C10H12O4	177,122,77
56.	Salicylic acid	8.58	137.0244	0.00	C7H6O3	108,81
57.	Methyl gallate	8.72	183.0295	-2.17	C8H8O5	137,123,
58.	Caffeic acid ethyl ester	8.88	207.0651	-5.71	C11H12O4	179,165,119,107,91
59.	Dimethoxy-benzoic acid	8.91	181.0505	-0.55	C9H10O4	153,109,95
60.	Coumaroylquinic acid	8.96	337.0937	2.40	C16H18O8	301,185,179,165
61.	Hydroxybenzoic acid	8.99	137.0250	4.37	C7H6O3	108,81
62.	Caffeic acid	9.09	179.0333	-8.93	C9H8O4	135,99,77
63.	Salicylic acid	9.13	137.0245	0.72	C7H6O3	122,93
64.	Coumaroylquinic acid	9.16	337.0893	10.65	C16H18O8	301,253,185,179,164,151,117
65.	Hydroxy-phenylacetic acid	9.32	151.0406	3.97	C8H8O3	136,108
66.	Ellagic acid	9.38	300.9996	2.03	C14H6O8	283,257,241,229,189,179
67.	Ferulic acid	9.41	193.0495	-5.86	C10H10O4	149,121,93,65
68.	Caffeic acid	9.53	179.0348	-0.55	C9H8O4	135,107,77
69.	Ferulic acid	10.25	193.0504	-1.03	C10H10O4	149,121,65
70.	Sinapic acid	11.76	223.0606	-2.24	C11H12O5	177,149,135
71.	Verbascoside	12.16	623.1972	-1.44	C29H36O15	577,461,285,161,137,84
72.	Coumaric acid hexoside	12.29	325.0897	-9.53	C15H18O8	257,189,83
73.	4-O-Cafeoyl-shikimic acid	12.58	335.0793	6.26	C16H16O8	295,199,189,131,113,93
74.	Syringoylquinic acid	13.96	371.0979	1.27	C16H20O10	327,287,259,219,191,113
*Flavonoids and derivatives*						
75.	(Epi)catechin	1.43	289.0720	-1.03	C15H14O6	243,189,128,113,109
76.	(Epi)catechin gallate	5.55	441.0816	-2.54	C22H18O10	289,179,167,125
77.	6-Geranylnaringenin	5.72	407.1838	-6.38	C25H28O5	399,271,255
78.	Butin	6.51	271.0611	-0.36	C15H12O5	253,225,153,135
79.	Taxifolin	7.25	303.0509	-0.42	C15H12O7	259,219,191,153,113
80.	Phloretin	7.28	273.0773	1.66	C15H14O5	229,199
81.	Pinocembrin	8.10	255.0662	-0.32	C15H12O4	239,227,211,193,183
82.	(Epi)catechin	8.26	289.0720	0.82	C15H14O6	245,205,189,135,113
83.	Kampferol derivative	9.12	331.0823	-0.08	C17H16O7	285,257,229,217,177.152,124,121
84.	Cirsimaritin	9.24	313.0750	9.90	C17H14O6	298,283,269,203,189,125,117
85.	Naringenin	9.65	271.0615	1.12	C15H12O5	177,151,119,107
86.	Tricin or Jaceosidin	9.77	329.0667	0.07	C17H14O7	314,299,271
87.	kaempferide	9.88	299.0565	1.33	C16H12O6	284,219,177,147,109
88.	Apigenin	9.89	269.0450	-1.85	C15H10O5	241,207,183,155,125,109
89.	Naringenin	10.13	271.0595	-5.90	C15H12O5	253,215,151,119
90.	Naringenin isomer	10.24	271.0617	1.86	C15H12O5	253,215,151,119
91.	Taxifolin	10.41	303.0506	-1.41	C15H12O7	259,219,191,113
92.	Tricin or Jaceosidin isomer	10.78	329.0666	0.00	C17H14O7	299,271,227,161
93.	(Epi)catechin	11.03	289.0697	-7.13	C15H14O6	245,205,189,113
94.	(Iso)rhamnetin	11.13	315.0745	7.61	C13H16O9	300,271,247,189,161,151,137,109
95.	(Iso)rhamnetin isomer	11.20	315.0745	7.61	C13H16O9	300,271,247,189,161,151,137,109
96.	Luteolin	11.24	285.0407	1.05	C15H10O6	199,175,133
97.	(Iso)rhamnetin isomer	11.31	315.0520	3.17	C16H12O7	300,247,189,151,137
98.	(Iso)rhamnetin isomer	11.37	315.0521	3.49	C16H12O7	247,189,151
99.	(Epi)catechin	11.56	289.0720	1.03	C15H14O6	243,189,181,113
100.	Isorhamnetin	11.75	315.0500	-3.17	C16H12O7	300,247,189,151,107,94
101.	Quercetin	11.84	301.0352	-0.58	C15H10O7	245,179,151,121,107
102.	Luteolin	12.40	285.0409	1.75	C15H10O6	257,199,175,133
103.	Eriodictyol	12.78	287.0564	1.00	C15H12O6	219,151,113
104.	Isorhamnetin	13.38	315.0528	5.71	C16H12O7	300,247,189,163,77
105.	Kampferol	14.22	285.0399	-1.75	C15H10O6	251,228,184,133
106.	Kampferol isomer	14.79	285.0395	-3.15	C15H10O6	241,217,175,133,93
107.	Kampferol isomer	14.93	285.0391	-4.56	C15H10O6	241,151,133
108.	(Epi)Catechin 3-O-gallate	15.08	441.0816	-2.54	C22H18O10	395,305,289,191
109.	Myricetin	15.38	317.0328	7.91	C15H10O8	271,249,181,113
110.	Prenylnaringenin	15.40	339.1235	-0.88	C20H20O5	293,271,203,197,183,135
111.	Chrysoeriol or diosmetin	15.64	299.0561	-0.04	C16H12O6	284,175,163,139
112.	Dihydroxy-trimethoxyflavone	15.94	343.0810	-3.86	C18H16O7	315,297,259,191,177
113.	Quercetin	16.13	301.0352	-0.58	C15H10O7	233,179,151,121
114.	Isorhamnetin	16.16	315.0513	0.95	C16H12O7	300,247,189,179,161,95,65
115.	(Tetrahydroxyflavone) Kampferol	16.47	285.0399	-1.75	C15H10O6	239,212,184,133
116.	(Tetrahydroxyflavone) Kampferol	16.78	285.0407	1.05	C15H10O6	239,212,175,133
117.	Phaseoloidin	16.81	329.0878	-0.2	C14H18O9	285,
118.	(Epi)Catechin	17.30	289.0710	-2.42	C15H14O6	221,181,153,113
119.	(Epi)Catechin	18.39	289.0716	-0.34	C15H14O6	221,181,113
120.	(Epi)Catechin	18.61	289.0724	2.42	C15H14O6	221,181,113
121.	Quercetin	21.71	301.0377	7.97	C15H10O7	286,255,179,151
*Fatty acids and derivatives*						
122.	Methylglutaric acid	2.39	145.0515	6.20	C6H10O4	101,61,57
123.	Octadecanedioic acid	10.72	313.2374	-3.29	C18H34O4	271,189,125
124.	Hydroxylinolenic acid	12.05	293.2129	2.33	C18H30O3	275,257,249,209,181,103
125.	Hydroxy octadecenoic acid	13.13	297.2430	-1.74	C18H34O3	253,223,183
126.	Hydroxyoctadecadienoic acid	13.57	295.2284	2.03	C18H32O3	277,189,159,115,103
127.	Hydroxy octadecenoic acid	14.03	297.2436	0.33	C18H34O3	253,225,185,145,105
128.	Hydroxy-octadecatrienoic acid	14.12	293.2121	-0.34	C18H30O3	209,181,103,97,57
129.	Octadecanoic acid, 2-hydro-1,3-propanediyl ester	14.34	571.2854	2.31	C39H40O4	315,255,241,153
130.	Lauric acid	14.47	199.1704	0.23	C12H24O2	155,129,69
131.	Decanoic acid	15.77	171.1395	2.61	C20H22O8	127,103
132.	Octadecatrienoic acid	19.28	277.2174	0.35	C18H30O2	249,209,163,145,103
133.	Hexadecenoic acid (palmitoleic acid)	20.60	253.2176	1.17	C16H30O2	---
134.	Hexadecanoic acid (palmitic acid)	20.68	255.2320	-3.74	C16H32O2	111,103
135.	Octadecadienoic acid (linoleic)	21.36	279.2326	-1.27	C18H32O2	233,103
136.	Methyl hexadecenoate	21.96	267.2330	1.17	C17H32O2	249,224,113
137.	Hexadecanoic acid (palmitic acid)	22.99	255.2329	-0.21	C16H32O2	---
138.	Stearic acid	23.56	283.2641	-0.54	C18H36O2	239,227,211,183
139.	Oleic acid	23.58	281.2484	-0.73	C18H34O2	239,227,211,183
140.	Methyl hexadecanoate	24.15	269.2487	0.36	C17H34O2	----
141.	Methyl octadecenoic acid (methyl oleate)	25.39	295.2646	1.17	C19H36O2	231,159,103
142.	Hydroxy-stearic acid	25.63	299.2568	-7.68	C18H36O3	284,231,163,105,95
143.	Hexadecanoic acid (palmitic acid)	26.05	255.2326	-1.17	C16H32O2	212,171,111,103
*Coumarins and derivatives*						
144.	Hydroxy methylcoumarin	2.49	175.0400	-0.39	C10H8O3	137,131,87
145.	Esculetin	3.18	177.0205	6.60	C9H6O4	149,121,77
146.	Esculetin	3.69	177.0200	3.77	C9H6O4	149,121,77
147.	Hydroxy-methylcoumarin	4.67	175.0400	-0.39	C10H8O3	147,131,118,87
148.	Esculetin	5.50	177.0192	-0.56	C9H6O4	133,121,89,87
149.	Brevifolin carboxylic acid	6.03	291.0143	-1.17	C13H8O8	247,189,161
150.	Hydroxycoumarin	6.32	161.0251	4.34	C9H6O3	133,117,76
151.	Brevifolin carboxylic acid	6.75	291.0158	3.98	C13H8O8	247,223,183,175
152.	Brevifolin carboxylic acid	11.27	291.0159	4.33	C13H8O8	247,223,179,161
153.	Methyl brevifolincarboxylate	17.00	305.0307	1.34	C14H10O8	273,245,221,217,187,113
Stilbene						
154.	Resveratrol	9.69	227.0716	1.32	C14H12O3	185,159,115,101,71
155.	Tetrahydroxystilbene	11.36	243.0655	-3.22	C14H12O4	200,175,171,131
156.	Tetrahydroxystilbene	11.47	243.0655	-3.22	C14H12O4	200,175,171,131
157.	Resveratrol	11.99	227.0694	1.32	C14H12O3	191,185,159,115,101,71
*Other detected compounds*						
158.	Catechol	1.18	109.0294	-0.95	C6H6O2	91,
159.	Hexose Sugar alcohol	1.23	181.0715	-1.10	C6H14O6	163,101,89,71,59
160.	Hexose sugar	1.48	179.0577	8.93	C6H12O6	161,135,99,95,77,59
161.	Quinic acid	2.80	191.0557	-2.16	C7H12O6	147,133,111
162.	Abscisic acid	3.72	263.1288	-0.32	C15H20O4	219,204,189, 177,157
163.	Quinic acid	3.94	191.0562	0.46	C7H12O6	147,133,111
164.	Estrone acetate	14.04	311.1683	9.96	C20H24O3	183,175,131
165.	Sphingolipid conjugate IV	18.79	566.3435	-4.94	C27H54NO9P	506,415,347,281
166.	Acyl glycerol	19.27	489.3018	-9.60	C25H46O9	443,367,281,193,161,113
167.	Unknown steroid	20.17	521.3113	-1.33	C29H46O8	447,385,317,249,181,175,113
168.	Unknown	21.54	399.1520	3.00	C15H28O12	331,245,195,177,148,113
169.	Oleanolic acid	22.07	455.3509	-4.61	C30H48O3	409,391,113

By comparing the retention times and MS data (including precise mass, isotopic distribution, and fragmentation pattern in negative ion mode) of the identified compounds with reference literature and making use of the digital natural product databases METLIN and RIKEN, the identification of metabolites was achieved.

### Phenolic acids and derivatives

Phenolic acids were the most prevalent class of metabolites in the date palm seed sample under investigation. In this inquiry, a total of sixty-six phenolic acids were tentatively found. Gallic, protocatechuic, caffeic, and ferulic acids were the main phenolic components (Fig. 1; Table 1). Together with the MS2 fragmentation, the metabolites were annotated using the M-H-ion obtained. For example, peaks 31 and 44 (Rt 11.33 min) indicated the loss of glucose and CO2 moieties, respectively, and were consequently classified as galloyl hexoside (Fig. 1; Table 1), with a precursor ion at m/z 331 and ms2 at m/z 169 (galloyl) [M-H-162]- and 125 [M-H-162-44].

Based on the detected precursor mass and the fragmentation pattern displaying product ions at m/z 191 and179 indicating the presence of quinic and caffeic acids, respectively, five isomers of caffeoyl quinic acid with the molecular formula C16H18O9 and m/z 353 were characterized at peaks 20, 26, 27, 29 and 30. The presence of ellagic acid was demonstrated by the molecular ion m/z 300.998 in two peaks, 33 and 39.

### Flavonoids and derivatives

According to UHPLC-MS analysis, flavonoids and their derivatives were found in date palm seeds after phenolic acids. According to [31], flavonoids are a prominent family of organic chemicals that are specifically categorized as secondary metabolites of plants that have a polyphenolic structure. Forty-seven flavonoids belonging to different groups were tentatively identified. Flavonols (kaempferol, isorhamnetin, and quercetin), flavones (apigenin and luteolin), and flavanols (catechin or epicatechin) are among the flavonoids that were examined.

A molecular ion with the formula C15H14O6 was found at m/z 289.07 by seven isomers (75, 82, 93, 99, 118, 119, and 120). Furthermore, the molecular ion [M-H]- at m/z 441.0816 was found at retention times of 5.55 and 15.08. The precursor ion at m/z 289, which is comparable to (epi)catechin, appeared following the galloyl moiety’s removal [M‒H‒152]. Therefore, it was decided to annotate chemicals 76 and 108 as (Epi)catechin gallate. A possible identification of seven (Iso)rhamnetin isomers (peaks 94, 95, 97, 98, 100, 104, and 114) was made at ESI − m/z 315.0745, with the product ion located at m/z 300.0269. Apart from some of their isomers, date palm seeds have also been found to contain several other aglycones, including butin (trihydroxyflavanone), taxifolin, phloretin, pinocembrin, cirsimaritin, naringenin, tricin or jaceosidin, kaempferide, apigenin, luteolin, quercetin, eriodictyol, Kampferol (tetrahydroxyflavone), myricetin, prenylnaringenin, chrysoeriol or diosmetin, dihydroxy-trimethoxyflavone, quercetin, and phaseoloidin (Fig. 1; Table 1).

### Fatty acids and derivatives

The date palm seeds under investigation contained twenty-two fatty acids and their derivatives. Several unsaturated fatty acids were tentatively identified from their mass spectra, as shown in Table 1. These included octadecatrienoic acid, palmitoleic acid (hexadecenoic acid), linoleic acid (octadecadienoic acid), oleic acid, and methyl octadecenoic acid (methyl oleate). The following saturated fatty acids were also shown: lauric acid, palmitic, stearic, octadecanedioic, and Methylglutaric acid.

Water molecules were lost in the illustrations of common hydroxy fatty acids, namely hydroxylinolenic, hydroxy octadecenoic, hydroxy-octadecadienoic, hydroxy-octadecatrienoic, and hydroxy-stearic acids.

### Organic acids

The roasted date palm seeds were found to contain seven different organic acids. They were all marked not only by the loss of 44 Da at MS2 fragmentation but also by their M-H‒ ions. They were identified as citramalic, cinnamic, itaconic, succinic, and glyceric acids. Together with cinnamoyl hexose (peak 7), there is also an organic hexoside that shows the loss of glucose and CO2 moieties, with a precursor ion at m/z 309.0987 and MS2 at m/z 147 and 103 [M-H-162-44] (Fig. 1; Table 1).

### Coumarins, isocoumarins and derivatives

In the material under examination, six coumarins and their derivatives were found, together with their isomers. These mostly consisted of esculetin and its isomers (peaks 145, 146, 148) that were determined by [M-H] ‒ at 177.02 and the product ion at 133, as well as hydroxycoumarin and hydroxy-methylcoumarin [M-H] ‒ at 161.0251 and 75.0400. Apart from the isocoumarins, methyl brevifolincarboxylate (peak 153) and its isomers (peaks 149, 151, and 152) are also present. These are indicated by [M-H] at 291.0158 and 305.0307.

### Other detected metabolites

In addition to their isomers, four stilbenes were found: tetrahydroxystilbene at [M-H] ‒ 243.07 and resveratrol at [M-H] − 227.07. Quinic acid and its isomer at [M-H] ‒ 191.056 classified as cyclitols, a triterpenoid oleanolic acid 169 with [M-H] ‒ at m/z 455.3509, a benzenediol, specifically catechol (Rt 1.18), a hexose sugar alcohol, and a hexose sugar in addition to abscisic acid classified as sesquiterpenoid plant hormone. Estrone acetate, a steroid aglycone with the chemical formula C20H24O3, was found at m/z 311.1683. It also has distinctive ions at m/z 183 and 145 [32]. Along with a sphingolipid conjugate with the chemical formula C27H54NO9P and [M-H] ‒ at 566.3435, which reveals characteristic mass pieces at 506 and 281 due to the loss of the phospholipid-specific 60 Da trimethylammonium group and 285 Da sphingosine group. In addition, it was found that there was an acyl glycerol, an unknown steroid, and an unknown.

### Sperm analysis

**Fig. 2:**
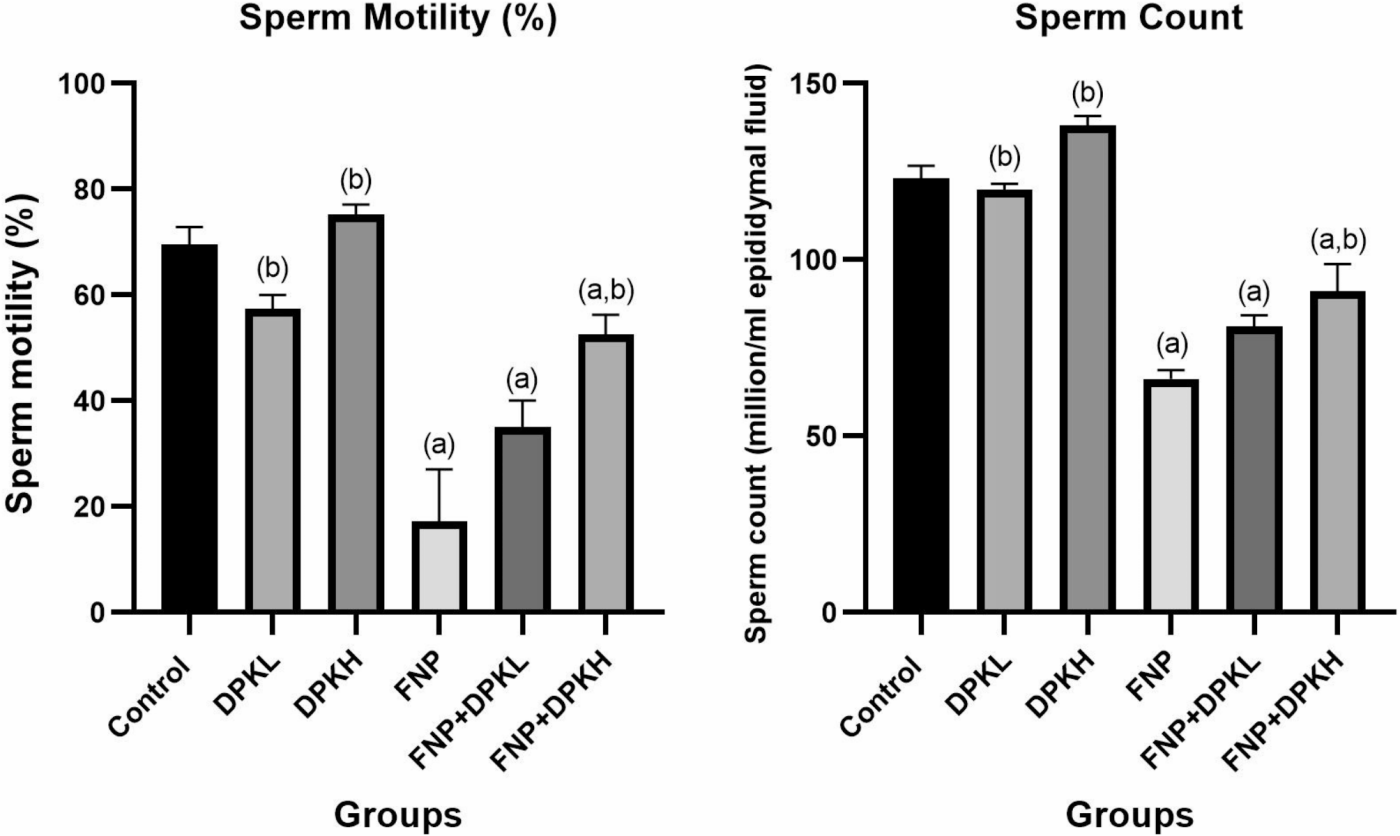
Sperm motility (%) and sperm count (10^6^ /ml epididymal fluid) in different groups. Data are represented as mean ± SEM. ^(**a**)^ Indicates significant difference from the corresponding control group at *P* ≤ 0.05. ^(**b**)^ Indicates significant difference from the corresponding FNP group at *P* ≤ 0.05. Abbreviations: FNP, Fenpropathrin; DPKL, Date Palm Kernel 200 mg/kg.bw; DPKH, Date Palm Kernel 400 mg/kg.bw

When compared to the rat control group, the animals that received FNP treatment showed a statistically significant drop (*P* < 0.05) in several sperm parameters, including motility and count. In comparison to the group of rats inebriated with FNP alone, the rats treated with DPK extract showed a significant improvement in sperm quality. (Fig. 2)

### Serum testosterone

**Fig. 3:**
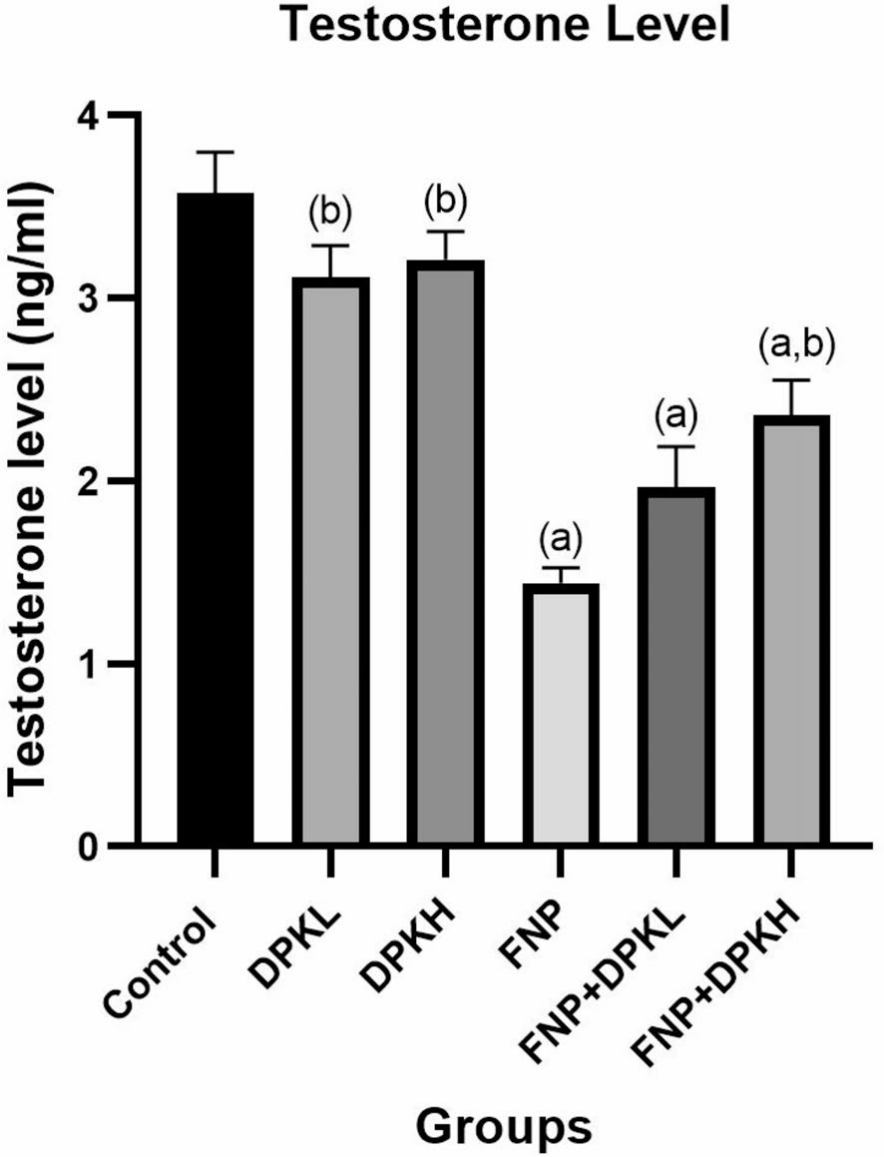
Serum testosterone in different groups. Data are represented as mean ± SEM. (**a**) Indicates significant difference from the corresponding control group at *P* ≤ 0.05. (**b**) Indicates significant difference from the corresponding FNP group at *P* ≤ 0.05. Abbreviations: FNP, Fenpropathrin; DPKL, Date Palm Kernel 200 mg/kg.bw; DPKH, Date Palm Kernel 400 mg/kg.bw

Serum testosterone levels were significantly lower in rats treated with FNP than in the control group. When compared to the group treated with FNP, the rats co-treated with DPK extract, at either a high or low dose, showed a statistically significant increase in serum testosterone levels (*P* ≤ 0.05) (Fig. 3).

### Oxidative stress markers

**Fig. 4:**
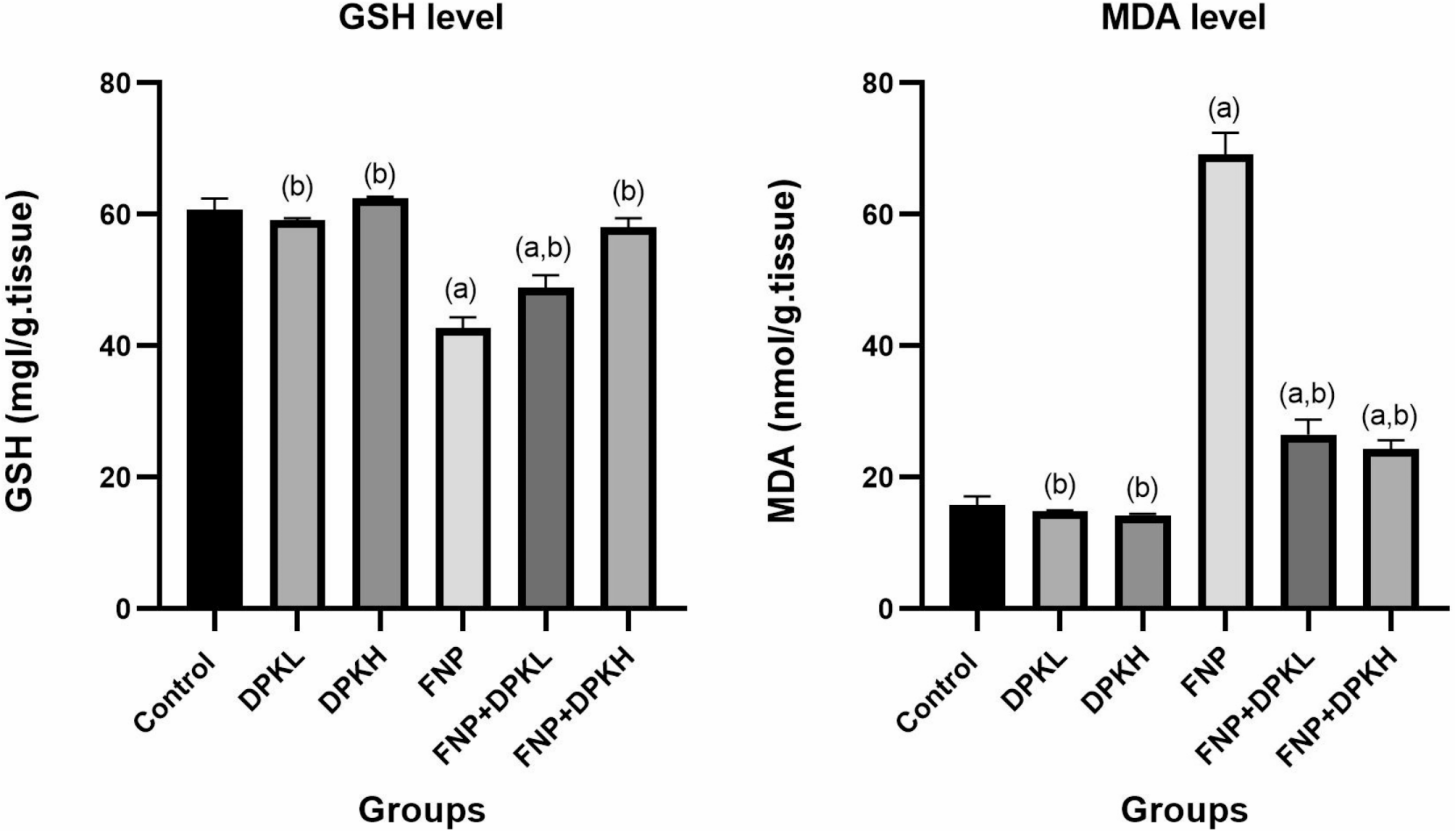
Tissue level of GSH and MDA in different groups. Data are represented as mean ± SEM. ^(a)^ Indicates significant difference from the corresponding control group at *P* ≤ 0.05. ^(b)^ Indicates significant difference from the corresponding FNP group at *P* ≤ 0.05. Abbreviations: GSH, glutathione; MDA, malondialdehyde; FNP, Fenpropathrin; DPKL, Date Palm Kernel 200 mg/kg.bw; DPKH, Date Palm Kernel 400 mg/kg.bw

It has been noted that prolonged and continuous oral exposure to FNP has a negative impact on the ratio of antioxidants to oxidants. A noteworthy increase (*P* ≤ 0.05) in malondialdehyde (MDA) and a reduction in reduced glutathione (GSH) levels when compared to a control group demonstrate these effects. Conversely, by increasing the levels of reduced glutathione (GSH) and decreasing the levels of malondialdehyde (MDA), the coadministration of DPK extract at both high and low dosages improved this balance (Fig. 4).

### Quantitative real-time PCR

#### The mRNA level of star gene

**Fig. 5:**
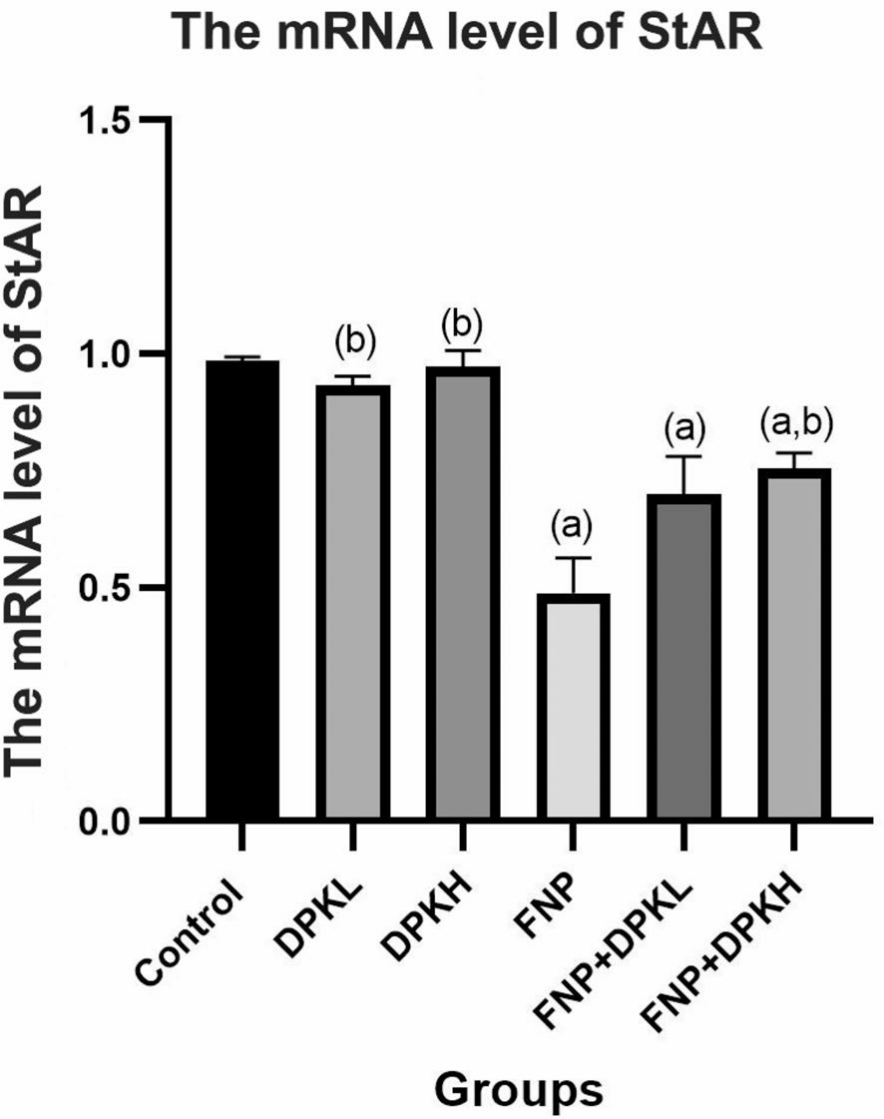
The mRNA level of StAR gene in different groups. Data are represented as mean ± SEM. ^(**a**)^ Indicates significant difference from the corresponding control group at *P* ≤ 0.05. ^(**b**)^ Indicates significant difference from the corresponding FNP group at *P* ≤ 0.05. Abbreviations: FNP, Fenpropathrin; DPKL, Date Palm Kernel 200 mg/kg.bw; DPKH, Date Palm Kernel 400 mg/kg.bw

When compared to the similar control group, the StAR mRNA level in the FNP-treated rats was significantly downregulated, with a p-value of less than 0.05. On the other hand, as evidenced by an increase in StAR mRNA level, rats who were also co-treated with DPK showed significant protection against fenpropathrin toxicity (Fig. 5).

#### Histopathology

**Fig. 6:**
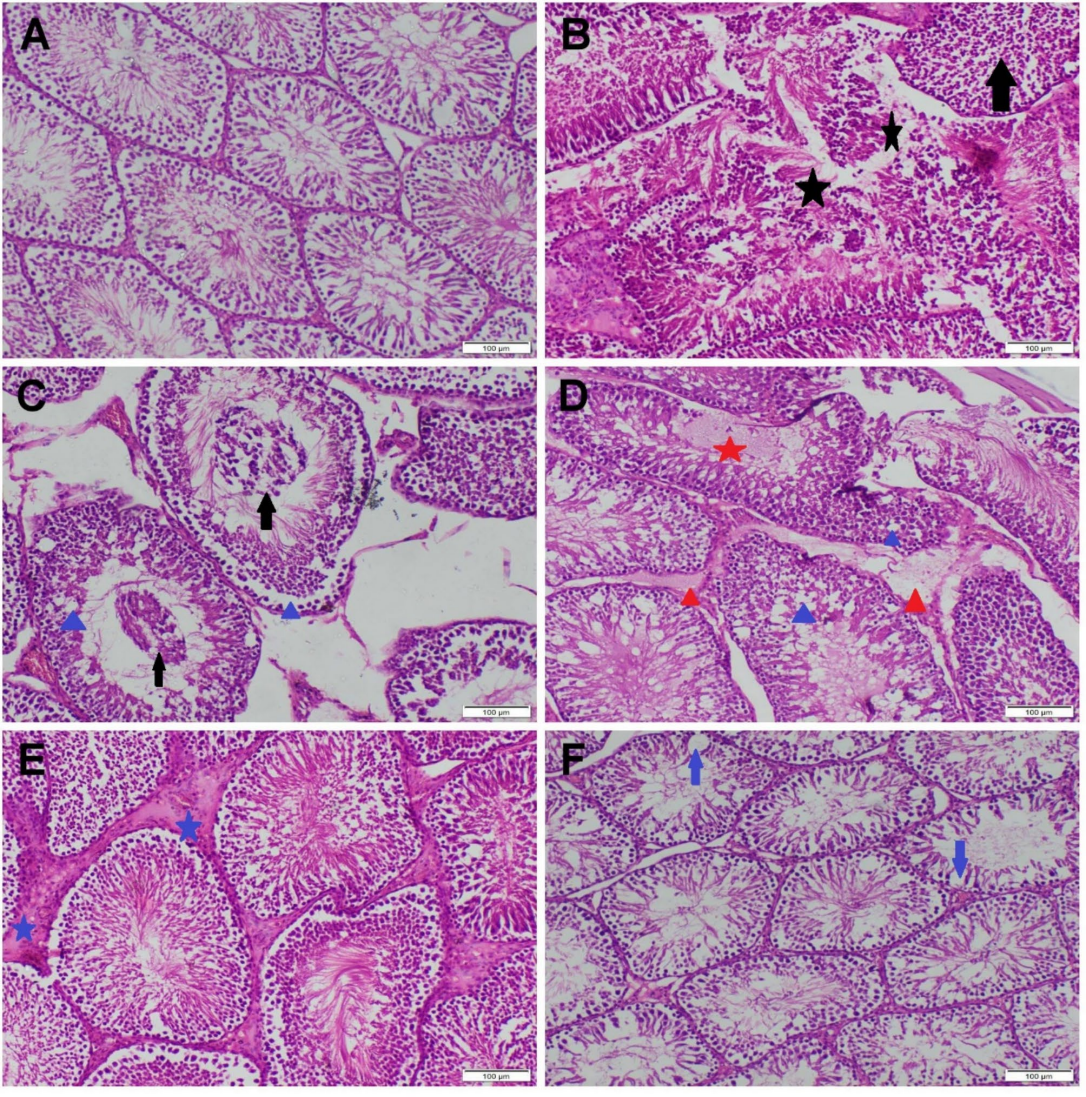
Photomicrographs of H&E stained testicular tissue sections of various groups. (**A**) The control group with normal histology. (**B**-**D**) FNP group displayed S.T. damage (black star), germ cell vacuolation (blue triangle), germ cell desquamation (black arrow), intratubular (red star), and intertubular edema (red triangle). (**E**) The FNP + DPKL group showed moderate Leydig cell hyperplasia (blue star). (**F**) The FNP + DPKH group exhibits mild germ cell loss (blue arrow) and vacuolations

Testicular tissue sections from the DPK receiving group and the control group displayed homogenous seminiferous tubules with different spermatogenic cell layers lining them in a typical histological arrangement (Fig. 6a). Only a small amount of interstitial tissue has whole Leydig cells in it. Conversely, the FNP-treated group showed significant histological alterations in every area. There was severe edema and total destruction to a few of the seminiferous tubules (Fig. 6b). Vacuolar degeneration, necrosis, and desquamation were observed by different spermatogenic cells (Fig. 6c). Most seminiferous tubules were convoluted; some shown evidence of spermatocyte and/or spermatogonia loss, while others were empty or had only one cell layer lining them. In most sections, deteriorating Leydig cells and tubular and interstitial edema were the major pathologies (Fig. 6d). The microscopic appearance of testicular sections improved in a dose-dependent manner in the groups who received both FNP and DPK. Sections of testicular tissue taken from rats given a low dose of DPK show that some of the spermatogenic cells lining the seminiferous tubules had individual cell necrosis. Leydig cell quantity increased mildly to moderately in most regions (Fig. 6e). Furthermore, all sections that received the high dose of DPK showed normal histological organization (Fig. 6f).

When comparing the FNP receiving group to the control group, the microscopic score of all parameters showed a substantial rise, according to the classical scoring. In contrast, the administration of DPK in addition to FNP reduces the microscopic score in a dose-dependent manner as compared to the FNP group (Table 2).

**Table 2:** Effect of FNP and/or DPK on the classical lesion scoring

	Control	DPKL	DPKH	FNP	FNP + DPKL	FNP + DPKH
GD/N	0 ^a^	0 ^a^	0 ^a^	4 ^d^	2 ^c^	1 ^b^
Desquamation	0 ^a^	0 ^a^	0 ^a^	4 ^c^	2 ^b^	0 ^a^
S.T atrophy	0 ^a^	0 ^a^	0 ^a^	3 ^c^	1 ^b^	0 ^a^
T edema	0 ^a^	0 ^a^	0 ^a^	3 ^c^	2 ^b^	0 ^a^
IS edema	0 ^a^	0 ^a^	0 ^a^	3 ^c^	2 ^b^	0 ^a^
LC mean score	3 ^b^	3 ^b^	3 ^b^	1 ^a^	5 ^c^	3 ^b^

Data is represented as median (*n* = 21 microscopic fields/group). Different superscript letters (a, b, c) indicate significant differences between groups at *P* < 0.05

#### Immunohistochemistry

**Fig. 7:**
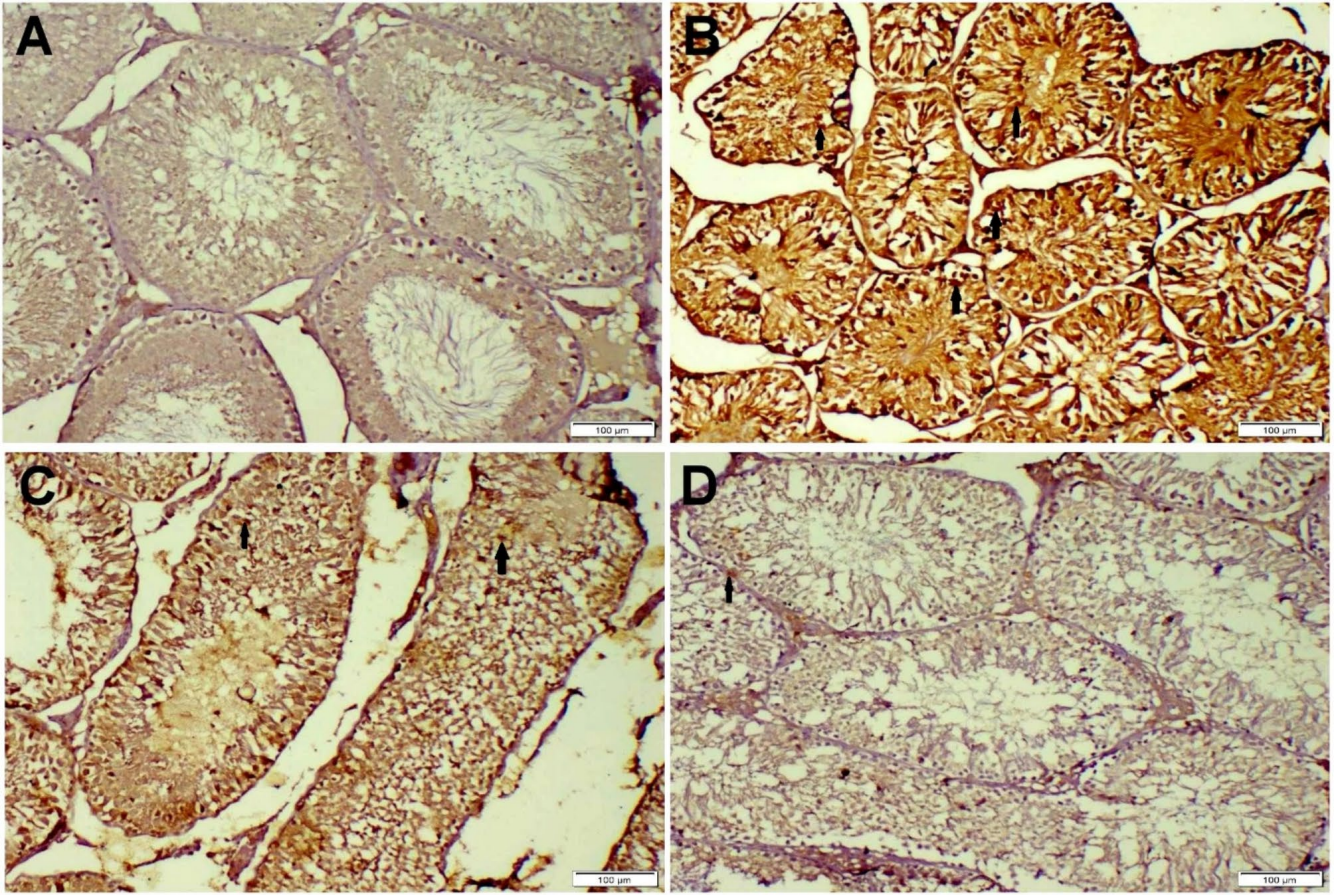
Photomicrographs representing casp-3 immunoexpression in various treatment groups. (**A**) Control group with normal baseline casp-3 protein expression. (**B**) FNP receiving group displayed strong casp-3 expression (arrow). (**C**) FNP + DPKL group displayed moderate casp-3 expression (arrow). (**D**) FNP + DPKH displayed weak casp-3 expression (arrow)

Testicular tissue slices from the FNP group showed high expression of the Caspase-3 protein throughout. However, the immunopositivity of such an apoptotic immunological marker decreased in treatment groups receiving DPKL in a dose-dependent manner. (Fig. 7).

## Discussion

Environmental pollutants cause hormonal alterations and malfunctioning testicles in humans and wildlife [33, 34]. Despite being recognized as environmental endocrine disruptors, pyrethroids have elicited concern over their potential adverse effects on the male reproductive system. This problem comes from the possibility that the testes, a target organ for many drugs and toxins, could be harmed. Hormone levels inside reproductive organs, including the testes, can be changed by injecting exogenous chemicals, as several research [35, 36] have shown. The reproductive endocrine system’s homeostasis may be disturbed by this perturbation. There may be a decrease in the quantity and number of sperm due to the harmful effects of medications and chemicals on spermatogenic cells. Frequent exposure to FNP is one of the ecological toxins that have a deleterious effect on testicular structure and function, resulting in a decrease in sperm and steroid hormone production [8]. Here, we measured blood testosterone levels, oxidative stress markers, sperm analysis, testicular microscopic appearance, caspase-3 expression, and StAR mRNA level in rats to assess the male reprotoxic effects of FNP. Furthermore, we provide a brief explanation of the putative mechanism behind DPK’s potential defense against testicular changes brought on by FNP.

Testicular toxicity mostly depends on the testes’ capacity to metabolize exogenous substances. Due to their high content of unsaturated fatty acids and exposure to low oxygen levels, the testes are particularly susceptible to oxidative damage [37]. In the present investigation, exposure to FNP dramatically reduced testosterone levels and sperm quality (motility and concentration). The result of this investigation is consistent with the findings of previous studies by Xiong et al. [38], which found that FNP, a form of pyrethroids (PYR), causes cell apoptosis in a dose- and time-dependent manner. Additionally, the results of Mohamed et al. [39] indicate that FNP poisoning modifies testes’ weight by inhibiting sperm production and steroidogenic enzymes and decreasing germ cells, elevating the proportion of defective sperm. A major oxidative issue arises when nontarget creatures, such as humans or laboratory animals, are routinely given sub-toxic amounts of PYRs, such as FNP. This could provide a reliable explanation for the notable changes in the quantity and motility of sperm in the rats treated with FNP. Furthermore, rats exposed to pyrethroid insecticide showed a significant drop in the number of sperm cells and an increase in the fraction of defective sperm, according to Osama et al. [40]. Furthermore, the drop in testosterone levels and sperm quality can be explained by the severe degeneration and loss of Sertoli cells, Leydig cells, and germinal epithelium in our histological results, as well as the atrophy of most seminiferous tubules.

Numerous hormones, such as luteinizing hormone (LH), follicle-stimulating hormone (FSH), gonadotropin-releasing hormone (GnRH), and sex steroids, are produced by the hypothalamic-pituitary-gonadal (HPG) axis. Reproductive outcomes are influenced by these hormones [41]. The testosterone hormone levels, significantly decreased in rats given FNP intragastrical at 4.7 mg/kg body weight for 60 days. There is strong evidence that pyrethroids have anti-androgenic characteristics, according to Castiello and Freire [42]. It is now clear that the androgen receptor (AR) regulated signalling pathway is important in this scenario. According to Yilmaz et al. [43], the data suggests that changes in testosterone synthesis, secretion, and distribution may also play a role in the mechanism of action of pyrethroids, in addition to their dependence on the androgen receptor (AR). It makes sense to believe that lower androgen levels would result in a lower sperm count, given the important function these hormones play in male fertility [44]. Exposure to FNP pyrethroid may inhibit the formation of androgens in the testes and their subsequent release and activity, resulting in an anti-androgenic impact.

The results of this study showed that FNP treatment administration decreased the levels of StAR mRNA. Previous research has shown a relationship between testicular injury and reduced expression of the StAR mRNA levels [45, 46]. Abd-Allah et al. [47] have linked structural defects in the testes to Leydig cell damage, which could be the mechanism responsible for the decrease in StAR protein expression. Steroidogenic genes were less expressed when FNP modulated the mRNA expression levels of transcription factors. The present study found a significant reduction in testosterone following a drop in StAR mRNA levels. This decrease is explained by the fact that StAR mRNA level is mostly found in steroidogenic tissues in vertebrates, which oversee the production of steroids [48].

The testes of rats treated with FNP showed a significant increase in oxidative stress indicators. According to several research [49–51], oxidative stress is a potent biomarker that is frequently used for PYRS toxicological assessment and may be the mechanism of action for its primary detrimental effects. Exposure to exogenous chemicals may result in an overabundance of free radicals, which can oxidatively damage biological components such as DNA, lipids, and proteins [52, 53]. Oxidative stress may be the cause of FNP-induced apoptosis. Since ROS levels were previously found to be much greater in groups who received FNP treatment [54, 55]. revealed that mitochondrial cytochrome P450 malfunctioned and lowered mitochondrial complex I activity, contributing to the oxidative stress brought on by pyrethroids.

The current study’s findings, which indicated that FNP significantly reduced GSH levels and increased MDA levels, were consistent with those of Nashed et al. [8] who found that FNP-exposed Wistar rats had significantly lower GSH levels and elevated MDA due to lipid peroxidation (LPO). Due to their lipophilic properties, which enable them to cross cellular membranes and result in LPO, Type II PYRs may cause oxidative damage [9]. According to Zeid et al. [5] a considerable increase in MDA concentration causes more ROS to be produced. Male rats exposed to lambda-cyhalothrin had significantly higher MDA levels, according to a prior study [56].

Rats given FNP had a range of abnormalities in their testes, which could be related to FNP-mediated oxidative stress, according to the latest results of histological research. Oxidative stress may cause sperm count reduction, hasten the death of germinal cells, and interfere with the gonads’ normal function and structural integrity. Rats subjected to synthetic pyrethroids have shown similar histological outcomes, according to other researchers [8, 39, 40]. Immunohistochemical staining was used to corroborate the reported histopathological changes, showing high expression of the casp-3 protein and apoptosis incidence. The progressive degradation of tissue functions and structure is mostly caused by apoptosis [57]. Excess reactive oxygen species facilitate calcium influx, activating apoptotic mechanisms. The translocation of cytosolic Bcl-2-associated X protein (Bax), a pro-apoptotic factor, to the mitochondria, together with the disruption of the Bax/B-cell lymphoma 2 (Bcl-2) ratio, an anti-apoptotic factor, produced by FNP, leads to caspase-3 activation [58]. The significant increase in caspase 3 protein expression in the FNP group suggests that testicular cells are more likely to undergo apoptosis. While the rats co-treated with DPK extract at either a high or low dose showed a statistically significant decrease in caspase 3 protein expression.

Biologically active substances called phytochemicals are produced by plants as they grow and develop. Some substances, including flavonoids, phenolics, fatty acids, and organic acids, play important roles in defending cells from damage and controlling genetic pathways [59, 60]. Numerous bioactive substances, such as phenolic acid, flavonoids, carotenoids, tocopherols, and carotenoids, are present in date seeds and have been shown to have therapeutic effects [61]. These ingredients’ anti-inflammatory, anti-hyperglycaemic, antioxidant, and anti-hyperlipidaemic qualities demonstrate their effectiveness. According to Kumar and Goel [62], phenolic compounds with a single carboxylic acid group are referred to as phenolic acids. Phenolic acids are divided into two classes based on the length of the carbon chain: hydroxycinnamic and hydroxybenzoic acids. Because phenolic acids can scavenge free radicals and increase the activity of enzymes that detoxify reactive oxygen species (ROS) and reactive nitrogen species (RNS), they help lessen the negative effects that these substances can cause [63].

Because DPK has a high antioxidant capacity, providing DPK extract with FNP improved sperm characteristics, hormone levels, and oxidative stress. In this investigation, oxidative stress, lipid peroxidation, and apoptosis were all successfully decreased in rats given DPK at the same time. This was achieved by lowering malondialdehyde (MDA) mediators and reactive oxygen species (ROS) while concurrently raising glutathione (GSH) levels. These substances may have antioxidant properties based on their suppression of oxidative stress markers.

The antioxidant activity, OH and O2 elimination capacity, propensity to chelate metal ions, and capacity to interact with other antioxidant metabolites are all linked to the pharmacological activities of flavonoids, which are essential parts of DPK. Via mechanisms like those involved in reducing oxidative stress, flavonoids have a protective effect on lipids. More precisely, the presence of a 3′4′-catechol group in the flavonoid B ring increases the effectiveness of the compound’s inhibition of lipid peroxidation. Two flavonoids with notable antioxidant qualities are kaempferol and epicatechin. They effectively eliminate free radicals and have a strong inhibitory effect on lipid peroxidation, according to Kumar and Pandey [64]. The present investigation found that DPK led to an increase in GSH levels and a decrease in MDA activity when compared to the group treated with FNP, which agrees with Zarie et al. [65], who reported that adult male Wistar rats exposed to DPK extract had significantly reduced MDA levels and increased GSH levels against High-fat diet group.

## Conclusion

The present study demonstrated that oral exposure to FNP for 60 days markedly impacted testicular tissue architecture, antioxidant levels, serum testosterone levels, and sperm characteristics. The obtained outcomes could be explained by the DNA-damaging characteristics of FNP and the mitochondrial trigger of apoptosis in rat spermatogonia cells. Additionally, the results showed that DPK, particularly at high dose levels, preserves rat testes from FNP-induced damage by reducing the reprotoxic effects of the drug through its potent antioxidant capacity and efficient antiapoptotic action.

## Data Availability

The authors confirm that the article contains the data needed to support the study’s findings.
